# Rapid detection of the *GJB2* c.235delC mutation based on CRISPR-Cas13a combined with lateral flow dipstick

**DOI:** 10.1515/biol-2025-1064

**Published:** 2025-03-11

**Authors:** Yueqin Deng, Juan Xu, Ming Yang, Yin Huang, Yifang Yang

**Affiliations:** Department of Otolaryngology Head and Neck Surgery, The Third Hospital of Changsha, Changsha 410015, Hunan, China

**Keywords:** CRISPR-Cas13a, lateral flow dipstick, *GJB2*, genetic testing, point-of-care testing

## Abstract

Hereditary hearing loss, an auditory neuropathy disorder, is characterized by its high prevalence and significant impact on the quality of life of those affected. In Chinese populations, the most prevalent gap junction beta-2 (*GJB2*) mutation hotspot is c.235delC. Currently available genetic tests require expensive instruments and specialized technicians or have long testing cycles and high costs, and therefore cannot meet point-of-care testing (POCT) requirements. The objective of this study was to evaluate the viability of a POCT kit. In only 42 min, we successfully identified the *GJB2* mutation site c.235delC by integrating CRISPR-Cas nucleic acid detection with recombinase-aided amplification (RAA) and a lateral flow dipstick (LFD) method. This method has the capacity to detect low-abundance nucleic acids (as low as 10^2^ copies/μL) and low mutation frequency (20%), in addition to accurately distinguishing wild-type, homozygous, and heterozygous mutation. This approach was utilized to assess blood samples from a total of 31 deaf patients and 5 healthy volunteers. All results were subsequently confirmed through the implementation of Sanger sequencing. Our detection results were consistent with Sanger sequencing results. The diagnostic sensitivity and specificity were 100%. The combination of CRISPR-Cas13a and LFD may be a promising method for POCT of deafness genes.

## Graphical abstract

**Figure:**
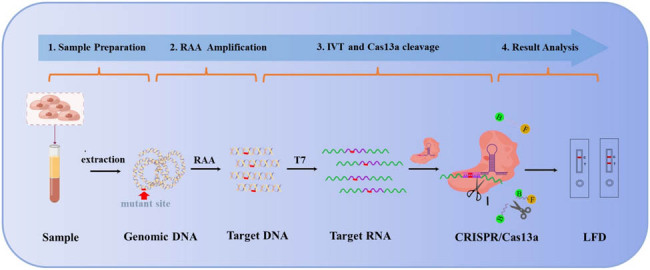

## 1 Introduction

The gap junction beta-2 (*GJB2*) is located on chromosome 13q11-q12 and encodes the connexin 26 (Cx26) [1]. *GJB2* mutation has been identified as a significant genetic factor contributing to non-syndromic hearing impairment, accounting for a range of 13–42% of cases [2,3]. In healthy populations, the carrier rate of *GJB2* mutation is estimated to be approximately 2.86% [4]. The most common mutation sites in *GJB2* include c.235delC, c.35delG, c.176_191del16, c.299_300delAT, etc. [5]. In Chinese populations, the most common *GJB2* mutation site is c.235delC, accounting for 77% of *GJB2* mutations [6].

As the most prevalent variant type for *GJB2*, missense variants have the capacity to disrupt the function of Cx26. Cx26 molecules have the ability to form gap junction (GJ) channels through extracellular docking. GJs facilitate intercellular communication through the diffusion of ions, metabolites, and small signaling molecules, which are imperative for the maintenance of normal function and fluid environment homeostasis of the inner ear. The primary pathogenic mechanisms of recessive pathogenic variants include trafficking defects, abnormal hemichannel activity, and abnormal GJ channel function [7]. These may result in potassium recycling disruption, adenosine-triphosphate-calcium signaling propagation disruption, and energy supply dysfunction, which lead to Cx26-related hearing loss [8]. The majority of patients with this type of deafness are congenital and have severe to profound hearing loss [9,10].

Early diagnosis and treatment are the most effective measures for controlling hereditary deafness. Current methods used to screen hereditary deafness genes include gene chip microarray [11], relevant fluorescence polymerase chain reaction (PCR) method [12], time-of-flight mass spectrometry [13], and next-generation sequencing [14]. However, currently available genetic tests require expensive instruments and specialized technicians or have long testing cycles and high costs and therefore cannot meet point-of-care testing (POCT) requirements. There is an urgent need for a rapid, accurate, and equipment-free detection method for deafness genes to better support clinical diagnosis and management of hereditary deafness.

The clustered regularly interspaced short palindromic repeat-CRISPR-associated protein (CRISPR-Cas) system has been demonstrated to not only stimulate a surge in gene editing [15] but also to result in advancements in molecular diagnostics. It has developed as a rapid, accurate, and portable nucleic acid detection technology [16,17]. The CRISPR-Cas system can also be used to detect gene mutations and single-nucleotide polymorphisms (SNPs) due to its ability to specifically recognize target nucleic acids [18]. Jin et al. [19] designed a fast and sensitive detection system for *SLC26A4* pathogenic mutations using CRISPR/Cas12a, but the results were fluorescence dependent, requiring a microplate reader for the detection, and the binding of the Cas12a-crRNA complex to the target DNA was limited by protospacer adjacent motif (PAM) sequences. However, the Cas13a protein does not require PAM sequences to recognize its targets, and the CRISPR-Cas13a system has significantly low off-target effects. Furthermore, the CRISPR-Cas13a system exhibits potential for SNP detection following the tuning of crRNAs [20].

The present study demonstrated the capacity to rapidly and accurately detect the *GJB2* mutation site c.235delC by combining CRISPR-Cas13a with RAA and lateral flow dipstick (LFD). This provides a novel and feasible strategy for the use of POCT for the detection of deafness genes.

## 2 Materials and methods

### 2.1 Materials

Oligonucleotides (primers), ssRNA-FB reporters, crRNAs, and PUC57 plasmids with customized gene sequences were synthesized by Sangon Biotech Co., Ltd (Shanghai, China) or purchased from the aforementioned company. NTP Mix, RNase inhibitor murine, and T7 RNA polymerase were purchased from New England Biolabs. The RAA Nucleic Acid Amplification Kit (Basic) was purchased from Hangzhou ZC Bio-Sci&Tech Co., Ltd (Hangzhou, China). The LwaCas13a protein was purchased from Nanjing Kingsley Biotechnology Co., Ltd (Nanjing, China). RNase Alert v2 was purchased from Thermo Fisher Scientific Inc. (Waltham, MA, USA). Nanogold (AuNPs) coated with rabbit anti-FAM antibody (IgG) was purchased from Xi’an Qiyue Biotechnology Co., Ltd (Xi’an, China).

### 2.2 RAA amplification of the target gene region

The design and synthesis of 30–32 bp RAA primers were conducted, with the ligation of the T7 promoter sequence for the T7 RNA polymerase (5′-AATTCTAATACGACTCACTATAG-3′) to the 5′ end of the forward primer (Table S1). The c.235delC of the *GJB2* mutation target was approximately 170–200 bp. RAA amplification was then performed using commercial RAA kits that had been fine-tuned according to the instructions. Specifically, 25 μL of A buffer, 2.5 μL of forward primer (10 μM), 2.5 μL of reverse primer (10 μM), 7.5 μL of RNase-free water, 10 μL of DNA template, and 2.5 μL of B buffer were added to each dry powder tube. After mixing, the reaction was incubated at 37°C for 20 min. The products of the RAA reaction were then removed for the cleavage assay and verified by electrophoresis on a 2% agarose gel.

### 2.3 Cas13a fluorescent cleavage assay

The CRISPR fluorescence reaction system was optimized with modifications to the concentrations of each reactant, as outlined in Kellner et al. [17]. The reaction system consisted of a total of 25 μL, including 1 μL rNTP mix (final concentration of 1 mM), 0.5 μL RNAase inhibitor (final concentration of 2 IU/μL), 0.5 μL LwaCas13a protein (final concentration of 20 nM), 0.25 μL T7 RNA polymerase (final concentration of 1 IU/μL), 0.25 μL MgCl2 (final concentration of 10 mM), 0.5 μL HEPES buffer (final concentration of 20 mM), 1.5 μL fluorescent reporter RNA (final concentration of 120 nM), 1.5 μL crRNA (final concentration of 120 nM), 5 μL RAA amplification product, and 14.25 μL RNase-free water. The above reaction solution was thoroughly mixed and placed in a fluorescence quantitative PCR instrument (excitation light/emission light wavelengths of 490 nm/520 nm, reaction temperature set at 37°C, fluorescence was collected every 2 min for a total of 15 times), and fluorescence intensity changes were recorded.

### 2.4 Preparation of lateral flow test strips

The lateral flow test strips comprised a sample pad, a gold standard pad, an absorbent pad, a PVC backing, a nitrocellulose membrane (NC membrane), a test line (T line), and a control line (C line). Initially, the gold standard pad was prepared by means of the supersaturated impregnation method. In a subsequent step, nanogolds (AuNPs) encapsulated with rabbit anti-FAM antibody (IgG) were resuspended in 1.5 mL of the embedding solution (0.25% TritonX-100, 8% sucrose, 20 mM Na3PO4, 5% bovine serum albumin). Thereafter, the AuNP solution was dripped onto the polyester membrane, which was then allowed to dry naturally, to prepare the gold standard pad. The NC membrane was then attached to the PCV base plate, containing T and C lines, which were sprayed with streptavidin (SA) (1 mg/mL) and mouse anti-rabbit IgG antibody (1 mg/mL), respectively. It should be noted that the T and C lines were 6 mm apart. The NC membrane was subjected to a drying process at a temperature of 37°C for a duration of 1 h. Subsequently, the sample pads, gold standard pads, and absorbent pads were affixed to the base plate, ensuring a 2 mm overlap, and then stored at 4°C in a dry environment.

### 2.5 Cas13a cleavage assay on lateral flow test strips

The CRISPR reaction system for test strip assays was adapted from the fluorescent CRISPR reaction system in the appropriate proportions. The reaction system thus consists of a total of 60 μL, including 2.4 μL rNTP mix (final concentration of 1 mM), 1.2 μL RNAase inhibitor (final concentration of 2 IU/μL), 1.2 μL LwaCas13a protein (final concentration of 20 nM), 0.6 μL T7 RNA polymerase (final concentration of 1 IU/μL), 0.6 μL MgCl2 (final concentration of 10 mM), 1.2 μL HEPES buffer (final concentration of 20 mM), 3 μL the reporter RNA for test strip assays (final concentration of 100 nM), 3.6 μL crRNA (final concentration of 120 nM), 5 μL RAA amplification product, and 41.2 μL RNase-free water. The above reaction system was thoroughly mixed and placed in a metal bath at 37°C for 20 min. Thereafter, all of the reaction products were then added to the sample wells of the CRISPR test strips. The results were then read after a period of 2 min had elapsed. The resultant data from the test strips were interpreted as follows: a negative result is indicated by the visibility of the T line (test line) and the C line (control line); conversely, a positive result is indicated by the disappearance of the T line and the visibility of the C line.

### 2.6 Clinical sample collection and DNA extraction

Blood samples were collected from 31 deaf patients and 5 healthy volunteers at the Third Hospital of Changsha. Genomic DNA was extracted from the blood samples using the Rapid Blood Genomic DNA Isolation Kit. The total yield of DNA extracted from the blood samples ranged between 40 and 250 ng/μL.


**Informed consent:** Informed consent has been obtained from all individuals included in this study.
**Ethical approval:** The research related to human use has been complied with all the relevant national regulations and institutional policies and in accordance with the tenets of the Helsinki Declaration and has been approved by the Institutional Ethics Committee of the Third Hospital of Changsha (KY-EC-2023-003).

### 2.7 Statistical analyses

Statistical analyses were conducted utilizing GraphPad Prism 9.5 software. The measurement data were expressed as the mean ± standard deviation. All experiments in this study were performed in triplicates. The diagnostic sensitivity and specificity were used to assess the performance of CRISPR-Cas13a combined with LFD against Sanger sequencing.

## 3 Results

### 3.1 The principle of the detection

**Figure 1:**
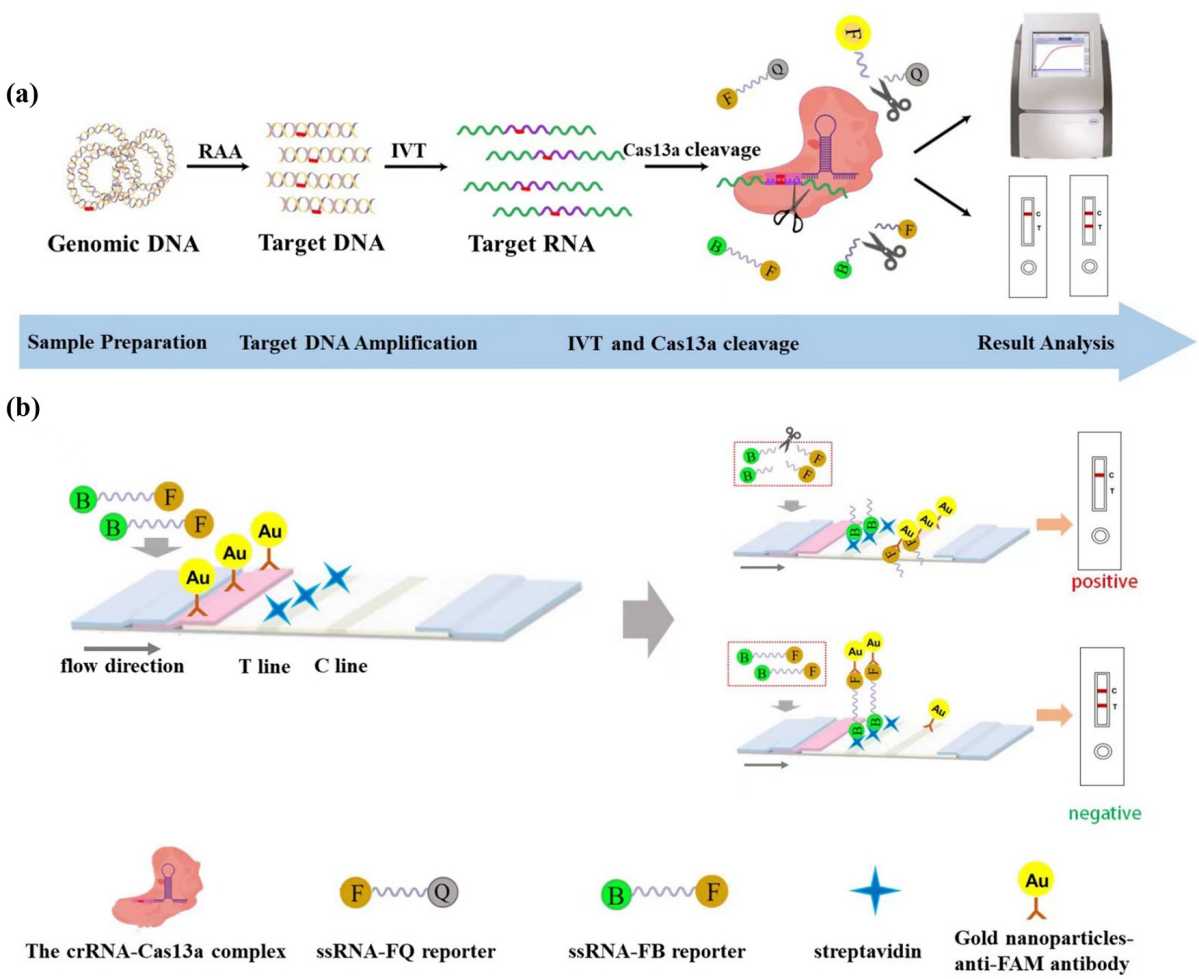
Illustration of the detection principle, which is based on the CRISPR/Cas13a system when combined with an LFD. (a) Schematic representation of a rapid *GJB2* detection method employing CRISPR/Cas13a. The workflow consists of sample preparation, target DNA amplification, TIV and Cas13a cleavage, and the result analysis. The ssRNA-FQ reporter and the ssRNA-FB reporter are cleaved when the crRNA is fully recognized by the target RNA, resulting in a fluorescent or chromatographic signal. RAA, recombinase aided amplification; IVT, *in vitro* transcription; F, fluorophore; Q, quencher; B, biotin. (b) The working principle of lateral flow test strips. The lateral flow test strip contains the test line (T line) and the control line (C line). The result of the test is considered positive if the T line disappears and the C line appears or negative if both the T and C lines are visible. F, fluorophore; B, biotin; Au, gold nanoparticles.

In this study, to distinguish the genotype of the *GJB2* c.235delC mutation site, dual crRNAs were introduced, i.e., wild-type crRNAs and mutant-type crRNAs. The workflow of the CRISPR-Cas13a molecular detection technology is illustrated in Figure 1a. This technology comprises target gene amplification, *in vitro* transcription of amplification products, Cas13a trans-cleavage, and result analysis. Specifically, the target gene was used to generate a large number of double-stranded DNA fragments containing the sequence of the locus to be detected using RAA isothermal amplification. The target RNA was then generated by *in vitro* transcription using T7 polymerase and was bound to the Cas13a-crRNA complex, which activated the trans-cleavage activity of the Cas13a protein on the ssRNA-FQ and ssRNA-FB reporters. The cleavage of these reporters, either ssRNA-FQ or ssRNA-FB, resulted in the production of fluorescent signals or lateral flow chromatographic changes.

The lateral flow strips based on CRISPR-Cas13a were colloidal gold test strips, the working principle of which is shown in Figure 1b. The test strips exhibited the following characteristics. The gold-labeled pad contained colloidal gold particles encapsulated with rabbit anti-FAM antibody; the test line (T line) was sprayed with SA; the control line (C line) was sprayed with mouse-anti-rabbit IgG antibody; and the ssRNA-FB reporter was a single-stranded RNA of approximately 22 nt in length, with the 5′ end modified with biotin and the 3′ end modified with FAM (Table S1). The trans-cleavage activity of the Cas13a protein was not activated when the crRNA failed to recognize the target RNA. The FAM group located at the 3′ end of the ssRNA-FB reporter gene has been demonstrated to bind to the rabbit anti-FAM antibody on the colloidal gold particles, thus forming a “biotin-FAM-anti-FAM immune complex” polymer. During the lateral flow chromatography process, a portion of colloidal gold bound to SA on the T line of the test strip with the “Biotin-FAM-anti-FAM antibody-colloidal gold” polymer formed a visible T line and another portion of colloidal gold that was not bound to the ssRNA-FB reporter on the test strip bound to the rabbit anti-FAM antibody on the colloidal gold particles to form a visible C line. In the presence of target RNA in the reaction system, Cas13a trans-cleavage activity was activated, and the ssRNA-FB reporter gene was cleaved, thus preventing the formation of the “biotin-FAM-anti-FAM immune complex” polymer cannot be formed. In the lateral flow chromatography, the result of the “T line disappears and C line appears” was due to the combination of all colloidal gold particles with mouse anti-rabbit IgG antibody in the C line through rabbit anti-FAM antibody.

### 3.2 Design and screening of RAA primers

**Figure 2:**
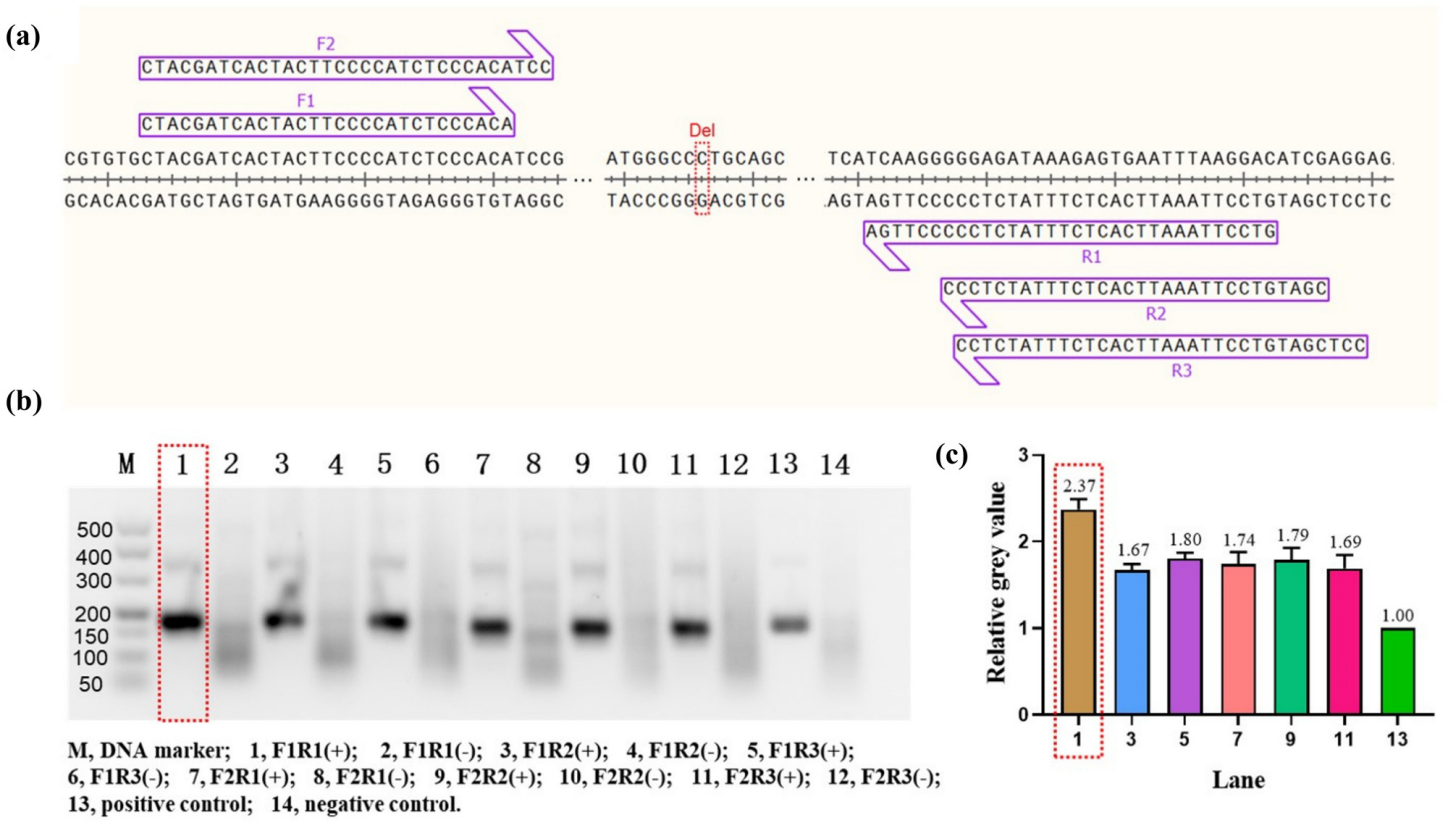
Design and screening of RAA primers. (a) Schematic representation of the RAA primers design. The red dashed rectangular box marks the c.235 locus of *GJB2* and “Del” is an abbreviation for deletion. F, forward primer; R, reverse primer. (b) Agarose gel electrophoresis analysis of RAA amplification products. The best results are marked by the red dashed rectangular box. “+” indicates that DNA template was added during RAA amplification. “–” indicates that RNase-free water was substituted during RAA amplification. (c) Gray value analysis of agarose gel electrophoresis bands. All bands were compared to the positive experimental group to form relative gray values. The best results are marked by the red dashed rectangular box. Error bars in Figure 2c present the standard deviation (SD) based on three independent gray value analyses.

As demonstrated in Figure 2a, two forward primers and three reverse primers were designed near the c.235 site of *GJB2*, respectively. Thereafter, one of the forward primers and one of the reverse primers were combined to form six pairs of primers. To evaluate the amplification performance of these six pairs of primers, wild-type DNA templates (Table S1) were amplified using the RAA Nucleic Acid Amplification Kit (Basic) and then analyzed by agarose gel electrophoresis. The experimental results are shown in Figure 2b, using primer combinations F1/R1, F1/R2, F1/R3, F2/R1, F2/R2, and F2/R3. The F1/R1 experimental group was found to be more effective. To ensure the reliability of the results, we also analyzed the gray scale values of each target band using ImageJ software, thus excluding the possibility of subjective visual errors. The outcomes are presented in Figure 2c, which illustrates the mean grey scale values of the amplification target bands for primer combinations F1/R1, F1/R2, F1/R3, F2/R1, F2/R2, and F2/R3 relative to the positive control. The mean gray scale values were 2.37, 1.67, 1.80, 1.74, 1.79, and 1.69, respectively, which further validates the hypothesis that the amplification efficiency of primer combination F1/R1 was optimal. (It should be noted that the influence of the length of the amplification products was negligible due to the almost similar size of the amplification products.)

### 3.3 Design and screening of crRNAs

**Figure 3:**
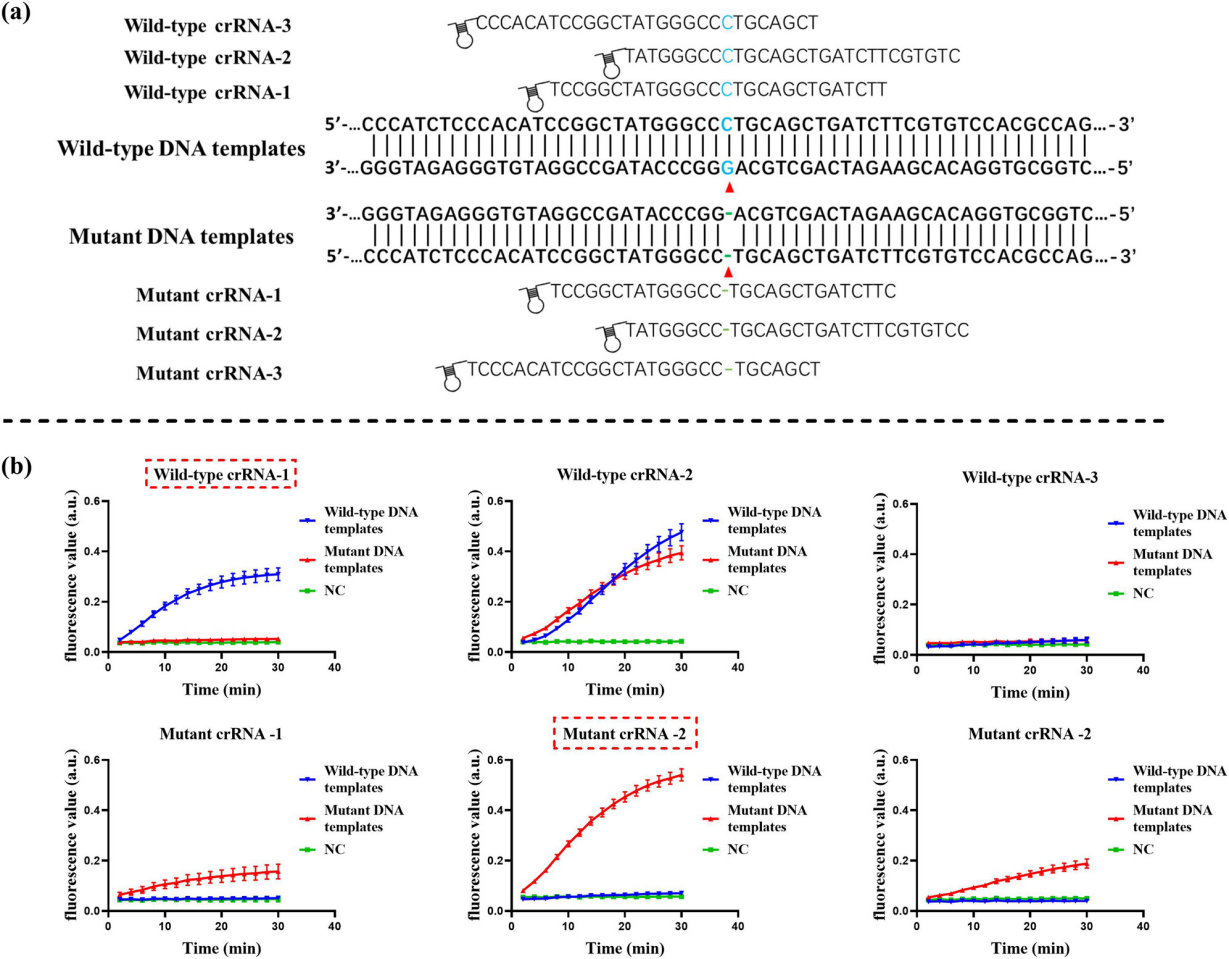
Design and screening of crRNAs. (a) Schematic representation of the crRNA design. The red triangles indicate the c.235delC mutation site of *GJB2*. (b) Screening of crRNAs by the CRISPR fluorescence-based assay. The best results are marked by the red dashed rectangular box. Error bars represent standard deviation (SD) from three independent tests.

The crRNA sequence of CRISPR-Cas13 system consists of a backbone region with a hairpin structure at the 5′ end and a spacer region that determines its specificity. In addition, it has been shown that there is a difference in the error tolerance of different base sites in the spacer sequence, which may affect the ability of CRISPR-Cas to detect SNP [21]. As shown in Figure 3a, we designed three crRNAs at the c.235 loci of *GJB2* for each wild-type DNA template and mutant DNA template and then compared the efficacy of crRNAs using the RAA-CRISPR fluorescence assay. The results are shown in Figure 3b. A strong fluorescence signal appeared when wild-type DNA templates were detected using wild-type crRNA-1, while there was no obvious fluorescence signal when mutant DNA templates were detected, which was comparable to the negative control. Meanwhile, a strong fluorescence signal appeared when mutant DNA templates were detected using mutant crRNA-2, while no obvious fluorescence signal appeared when wild-type DNA templates were detected, which was comparable to the negative control. All other crRNAs tested to detect wild-type or mutant DNA templates showed cross-reactivity or failed to achieve efficient detection performance, so none of them was an ideal choice. Therefore, we finally selected wild-type crRNA-1 and mutant crRNA-2 to form a dual-crRNA system for subsequent experiments because of their better specificity and efficient detection performance.

### 3.4 The detection analytical sensitivity

**Figure 4:**
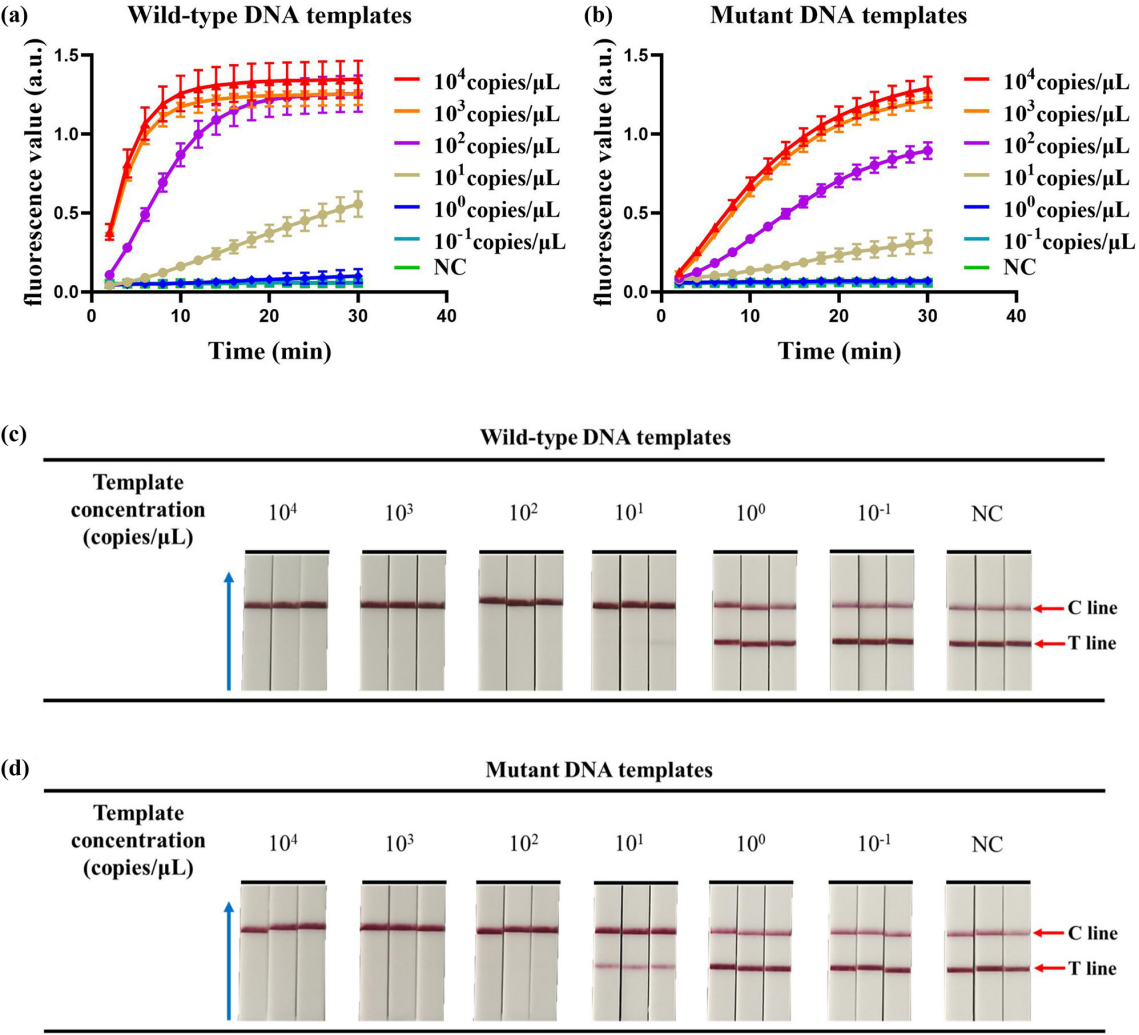
Detection of analytical sensitivity. (a) The detection sensitivity of wild-type DNA templates by the CRISPR fluorescence-based assay. (b) The detection sensitivity of mutant DNA templates by the CRISPR fluorescence-based assay. (c) The detection sensitivity of wild-type DNA templates by the CRISPR combined with LFD assay. (d) The detection sensitivity of mutant DNA templates by the CRISPR combined with LFD assay. The blue arrow points to the direction of flow of the test strip. NC, negative control. The error bars represent the standard deviation (SD) from three independent tests.

After optimizing crRNAs in the dual-crRNA detection system, the analytical sensitivity of the CRISPR fluorescence-based detection system and the CRISPR chromatography test strip-based detection system was tested using a plasmid DNA concentration gradient method. We diluted the wild-type and mutant plasmids to 10^4^, 10^3^, 10^2^, 10^1^, 10^0^, and 10^−1^ copies/µL, respectively, and used these two plasmids as templates for the CRISPR fluorescence-based assay and the CRISPR chromatography test strip-based assay to evaluate their detection sensitivity. As shown in Figure 4a and b, when the concentration of wild-type DNA template or mutant DNA template was greater than or equal to 10^1^ copies/μL, a strong fluorescence signal appeared using the dual crRNA detection system based on the CRISPR fluorescence assay, whereas no obvious fluorescence signal appeared when the concentration was less than 10^1^ copies/μL. Similarly, when the concentration of the wild-type DNA template or the mutant DNA template was greater than or equal to 10^2^ copies/μL, positive results appeared, and even when the concentration of wild-type DNA template was 10^1^ copies/μL, positive results were obtained in two out of three tests (Figure 4c and d). This high detection sensitivity is sufficient for the clinical detection of blood samples.

### 3.5 The ability to detect mutation frequencies

**Figure 5:**
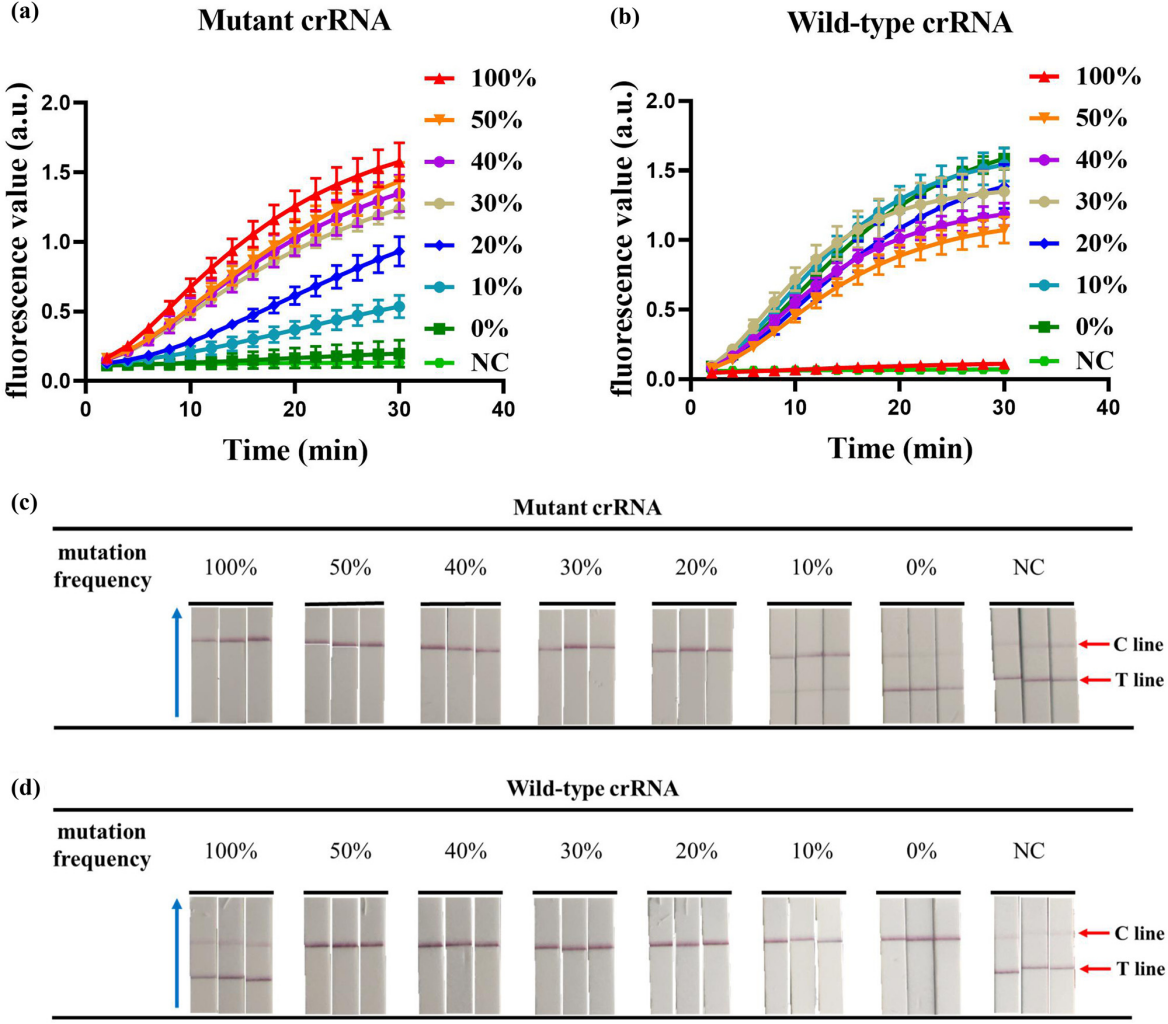
Detection of mutation frequency. (a) Detection of mutation frequency by the CRISPR fluorescence-based assay using mutant crRNA. (b) Detection of mutation frequency by the CRISPR fluorescence-based assay using wild-type crRNA. (c) Detection of mutation frequency by the CRISPR combined with LFD assay using mutant crRNA. (d) Detection of mutation frequency by the CRISPR combined with LFD assay using wild-type crRNA. The blue arrow points to the direction of flow of the test strip. NC, negative control. Error bars represent the standard deviation (SD) from three independent tests.

To evaluate the ability of this dual-crRNA detection system to identify mutant genotypes, we mixed wild-type DNA templates and mutant DNA templates at a concentration of 10^4^ copies/μL at 100, 50, 40, 30, 20, 10, and 0% mutation rates and then assayed them with CRISPR fluorescence-based and CRISPR chromatography strip-based assays, respectively. As shown in Figure 5a and b, when the mutation frequency was greater than or equal to 10%, the mutant crRNA detection system using CRISPR-based fluorescence detection showed a strong fluorescence signal, whereas when the mutation frequency was 100%, the wild-type crRNA detection system using CRISPR-based fluorescence detection did not show a significant fluorescence signal. Similarly, when the mutation frequency was higher than or equal to 20%, the mutant crRNA detection system using CRISPR chromatography strip-based assay showed a positive result, whereas when the mutation frequency was 100%, the wild-type crRNA detection system using CRISPR chromatography strip-based assay showed a negative result (Figure 5c and d). The above results indicate that the CRISPR detection system utilizing this dual-crRNA system can effectively distinguish mutant genotypes.

### 3.6 Clinical samples mutation detection

**Figure 6:**
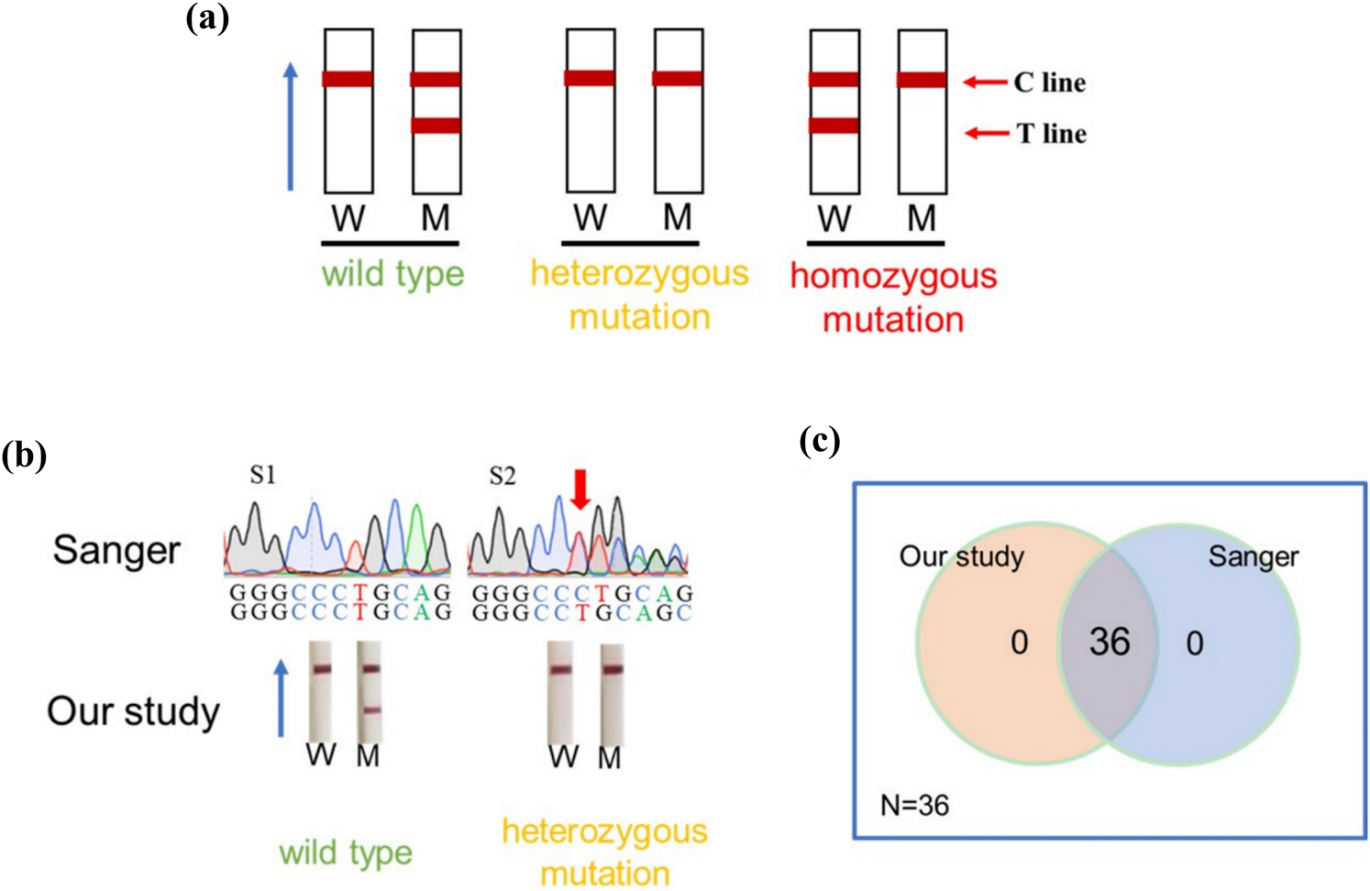
Detection of clinical samples. (a) The schematic diagram of allele discrimination by CRISPR-Cas13a combined with LFD. W indicates the LFD using wild-type crRNA; M indicates the LFD using mutant crRNA. (b) Representative results of clinical sample analyses using CRISPR-Cas13a combined with LFD and Sanger sequencing. Red arrows mark the c.235delC mutation site. (c) The Venn diagram shows the number of samples with consistent results using the CRISPR-Cas13a combined with LFD and the Sanger sequencing assay.

The c.235delC site was analyzed in blood samples from 31 deaf patients and 5 healthy volunteers using a CRISPR-Cas13a-based assay in combination with LFD and Sanger sequencing. Positive LFD result using wild-type crRNA and negative LFD result using mutant crRNA indicated that the genotype was wild type. Positive LFD result using wild-type crRNA and mutant crRNA indicated that the genotype was heterozygous mutation; Negative LFD result using wild-type crRNA and positive LFD result using mutant crRNA indicated that the genotype was homozygous mutation (Figure 6a). Blood samples from 6 deaf patients were tested, and the CRISPR chromatographic strip-based assay showed both heterozygous mutations (Figure 6b). CRISPR chromatography strip-based assay showed wild types in other samples including 25 deaf patients and 5 healthy volunteers (Figure 6b). We only showed representative results, and complete results were detailed in the supplementary material (Figure S1). The homozygous mutation was not available in our results due to a lack of sufficient clinical samples. At the same time, all samples were genotyped using Sanger sequencing. The results of the CRISPR-Cas13a combined with the LFD assay were 100% (36/36) consistent with the results of the Sanger sequencing assay (Figure 6c). Compared with the Sanger sequencing method, the diagnostic sensitivity and specificity of CRISPR-Cas13a combined with LFD were found to be 100% (6/[6 + 0]) and 100% (30/[0 + 30]), respectively (Table 1).

**Table 1:** Comparison of results between Sanger sequencing and CRISPR-Cas13a combined with LFD

CRISPR-Cas13a combined with LFD	Sanger sequencing	
	Heterozygous mutation	Wild type
Heterozygous mutation	6	0
Wild type	0	30

## 4 Discussion

The World Report on Hearing estimated that more than 1.5 billion people worldwide are affected by deafness. It is noteworthy that more than half of the observed cases of hearing impairment in newborns are attributable to genetic factors [22]. The implementation of widespread genetic screening and diagnosis of deafness in specific populations has the potential to reduce the birth of deaf children and is identified as a key factor in the early detection, diagnosis, and treatment of hereditary deafness. Currently, genetic testing for hereditary deafness is limited to specific genetic laboratories or experimental centers that are unable to meet POCT requirements of deafness genes and is generally expensive.

SNPs are major genetic targets for hearing loss screening. The most prevalent variant is *GJB2* c.235delC in the Chinese newborn population and East Asia [6]. CRISPR/Cas-mediated gene editing has received increasing attention as a prospective approach for modeling and treating hereditary deafness, although there are very few applications in the genetic diagnosis of deafness. The CRISPR-Cas13 system, which is characterized by its ability to undergo cis-cleavage and trans-cleavage, has been extensively and effectively utilized for the rapid and sensitive detection of nucleic acids. Feng Zhang’s team [16] developed a platform termed SHERLOCK (specific high-sensitivity enzymatic reporter unlocking) that utilized isothermal amplification and Cas13 to detect RNA or DNA mutations through lateral flow. The technology demonstrates its potential as a platform for rapid, portable, visual nucleic acid detection and instrument-free analysis. The ability to detect SNPs allows SHERLOCK to perform rapid and accurate allele typing. By combining CRISPR-Cas12a with isothermal amplification, Doudna et al. [23] developed a method called DNA endonuclease-targeted CRISPR *trans* reporter (DETECTR), which facilitates the rapid and specific detection of DNA. Nalefski et al. [24] examined the nucleic acid detection properties of Cas13 and Cas12a enzymes. Their findings revealed that the activation of Cas12a trans-activity was rate-limited by steps occurring after target binding. Conversely, the Cas13a RNase was rapidly activated following target binding. Moreover, CRISPR-Cas13a binding to target RNA does not require PAM sequences. In our study, a rapid and accurate assay for the c.235delC mutation site of *GJB2* was established by utilizing the powerful amplification performance of RAA, the efficient and specific discriminatory ability of the CRISPR-Cas13a system, and the simplicity and portability of the LFD. We introduced a dual-crRNA CRISPR system that significantly improved the sensitivity of the SNP genotyping including wild-type, homozygous and heterozygous mutation. The method is highly sensitive and can stably detect 10^2^ copies/μL of DNA plasmid template, which is sufficient for the detection of clinical samples. The entire detection process, including RAA isothermal amplification (39°C, 20 min), CRISPR cleavage (37°C, 20 min), and result presentation via LFD (room temperature, 2 min), took only 42 min in total and did not require costly instrumentation beyond a simple temperature control device. The results could be judged by naked eyes. We tested blood samples from 31 deaf patients and 5 healthy volunteers using this approach, including 30 wild types and 6 heterozygous mutations of c.235delC. The detection results were consistent with Sanger sequencing results. These results showed that it is feasible to detect deafness genes using CRISPR-Cas13a combined with RAA and LFD. It has been indicated that the SHERLOCK platform has the advantage of low cost compared to other platforms such as TaqMan qPCR. A typical single-plex reaction is estimated to cost as little as $0.60 [17,21].

Generally, detecting multiple target molecules in a single reaction is necessary for many applications. SHERLOCK exploited differences in the recognition of reporter probe sequences by different Cas13 homolog proteins and Cas12 to screen multiple orthogonal diagnostic systems for the detection of multiple targets [16]. Using real-time PCR and antibody-based lateral flow systems, Xu’s team devised a fast visual diagnostic system for the detection of multiple carbapenemase genes [25]. Cas13’s well-characterized preference to cleave polynucleotides renders it particularly well-suited for multiplexed assays [26]. The present study will focus on developing a multiplex assay that combines the most common deafness gene variants, which has considerable potential. The rapid development of CRISPR-based diagnostics (CRISPR-dx) technology has made it feasible to create multiplex lateral-flow assays with several test lines for distinct targets.

However, this method also has some limitations: first, this technique requires specific crRNA, which limits its ability to detect only known mutant genes, not unknown mutant genes. Furthermore, RAA isothermal amplification and CRISPR cleavage require two steps, along with many reaction components, which complicates the process. The overarching objective of this project is to formulate a method that can be executed in various healthcare settings with only a small thermostatic water bath and pipettes, and with simple training for operators. At the grassroots level, the lack of supplies and relevant knowledge among medical staff requires that all testing methods must be simple and low-cost. Due to the aforementioned limitations, our testing methods are currently incapable of meeting the grassroots community’s needs.

CRISPR-based diagnostics (CRISPR-Dx) is a promising technology for detecting nucleic acids while concomitantly reducing equipment requirements. We are optimistic about research into the rapid pretreatment of clinical samples, based on a large number of studies on cellular nucleic acid-free extraction from blood and saliva samples [27]. The assay, designated SHINE (streamlined highlighting of infections to navigate epidemics), has been shown to simplify the workflow by ambient sample lysis procedure and the lyophilization of test reagents, thereby enabling isothermal amplification and CRISPR-Cas13 detection in a single step [28]. We will focus on sample processing, nucleic acid amplification-free, multiple tests, and integrated simple detection devices, which will significantly improve the accessibility of diagnostic tests.

## 5 Conclusions

Our study demonstrated that this method is not only capable of detecting low abundance nucleic acids (as low as 10^2^ copies/μL) and low mutation frequency (20%) but also accurately distinguishes between wild-type, homozygous and heterozygous mutation without expensive instrumentation. This CRISPR-based lateral flow chromatography test strip provides a new viable solution for POCT of deafness gene mutation locus, which could be widely used in the future at the grassroots level, where there is a lack of technicians and equipment.

## Supplementary Material

Supplementary Material
