# Supplementary material for: Rapid detection of the GJB2 c.235delC mutation based on CRISPR-Cas13a combined with lateral flow dipstick
Source: Open Life Sci. 2025 Mar 11;20(1):20251064. doi: 10.1515/biol-2025-1064 (PMC11909576; doi:10.1515/biol-2025-1064)
Supplement: Supplementary Material [file biol-2025-1064-suppl.pdf]

Supplementary material

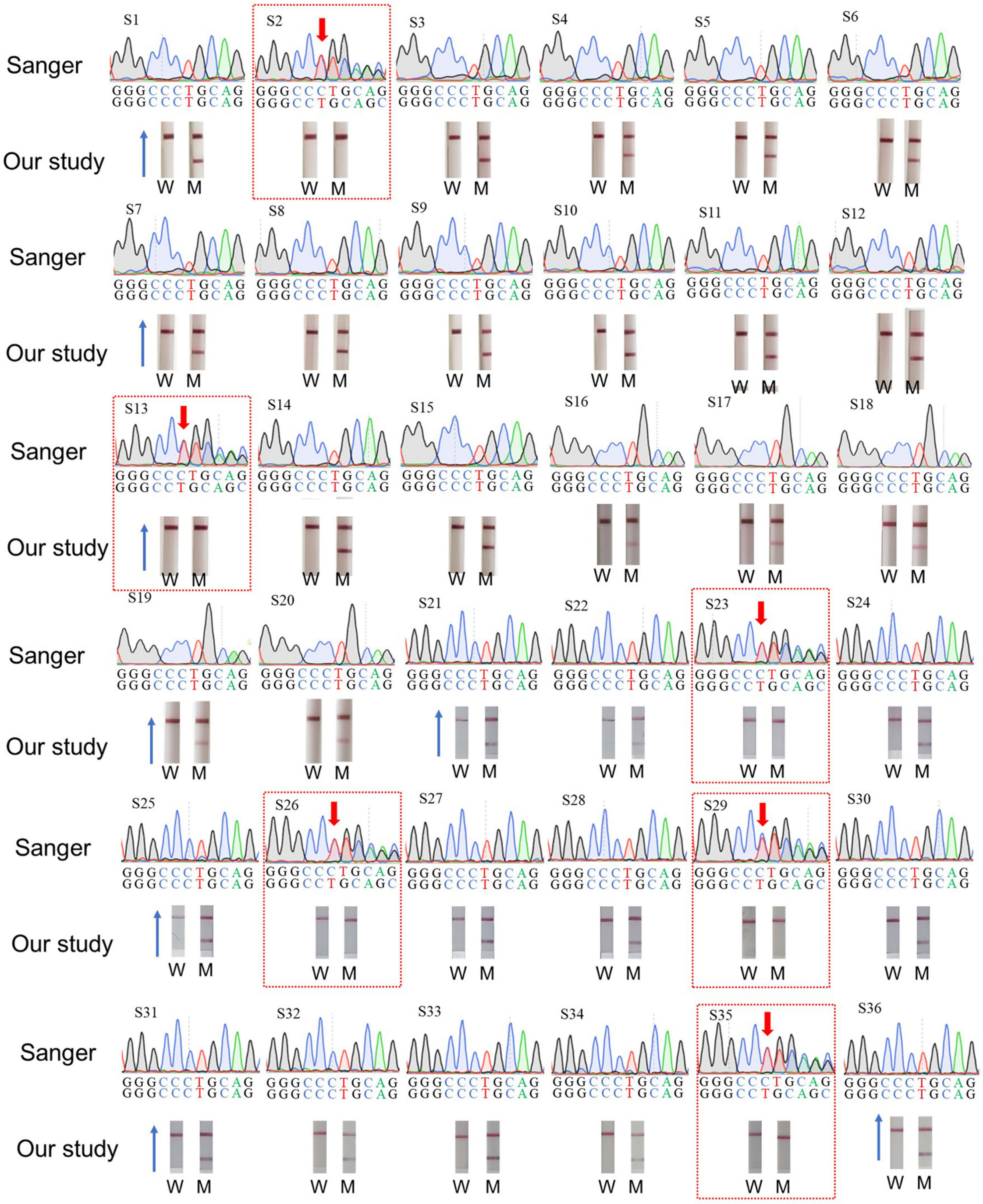

Figure S1: Analysis of clinical samples using CRISPR-Cas13a and Sanger sequencing methods. W indicates the LFD using wild-type crRNA; M indicates the LFD using mutant crRNA. Red arrows mark the c.235delC mutation site.

Table S1: The nucleotide sequences

Name	The nucleotide sequences (5′–3′)
Wild-type DNA template	AGCCAGGCTGCAAGAACGTGTGCTACGATCACTACTTCCCCATCTCCCACATCCGGCTATGGGCCCTGCAG CTGATCTTCGTGTCCACGCCAGCGCTCCTAGTGGCCATGCACGTGGCCTACCGGAGACATGAGAAGAAGA GGAAGTTCATCAAGGGGGAGATAAAGAGTGAATTTAAGGACATCGAGGAGATCAA
Mutant DNA template	AGCCAGGCTGCAAGAACGTGTGCTACGATCACTACTTCCCCATCTCCCACATCCGGCTATGGGCCTGCAGCT GATCTTCGTGTCCACGCCAGCGCTCCTAGTGGCCATGCACGTGGCCTACCGGAGACATGAGAAGAAGAGGA AGTTCATCAAGGGGGAGATAAAGAGTGAATTTAAGGACATCGAGGAGATCAA
RAA forward primer-1 (F1)	AATTCTAATACGACTCACTATAGGGCTACGATCACTACTTCCCCATCTCCCACA
RAA forward primer-2 (F2)	AATTCTAATACGACTCACTATAGGGCTACGATCACTACTTCCCCATCTCCCACATCC
RAA reverse primer-1 (R1)	GTCCTTAAATTCACTCTTTATCTCCCCCTTGA
RAA reverse primer 2 (R2)	CGATGTCCTTAAATTCACTCTTTATCTCCC
RAA reverse primer-3 (R3)	CCTCGATGTCCTTAAATTCACTCTTTATCTCC
Wild-type crRNA-1	GGGAUUUAGACUACCCCAAAAACGAAGGGGACUAAAACAAGAUCAGCUGCAGGGCCCAUAGCCGGA
Mutant crRNA-1	GGGAUUUAGACUACCCCAAAAACGAAGGGGACUAAAACGAAGAUCAGCUGCAGGCCCAUAGCCGGA
Wild-type crRNA-2	GGGAUUUAGACUACCCCAAAAACGAAGGGGACUAAAACGACACGAAGAUCAGCUGCAGGGCCCAUA
Mutant crRNA-2	GGGAUUUAGACUACCCCAAAAACGAAGGGGACUAAAACGGACACGAAGAUCAGCUGCAGGCCCAUA
Wild-type crRNA-3	GGGAUUUAGACUACCCCAAAAACGAAGGGGACUAAAACAGCUGCAGGGCCCAUAGCCGGAUGUGGG
Mutant crRNA- 3	GGGAUUUAGACUACCCCAAAAACGAAGGGGACUAAAACAGCUGCAGGCCCAUAGCCGGAUGUGGGA
ssRNA-FB reporter	5’-Biotin-TrUrUrUrUrUrUrUrUrUrUrUrUrUrUrUrUrUrUrC-FAM-3’

RAA primers, crRNA, reporter RNA-2, wild DNA template plasmid and mutant DNA template plasmid were synthesized by Sangon Biotech (Shanghai) Co., Ltd and the details of nucleic acid sequences are shown in Table 1.
